# “Remaining Vigilant” While “Enjoying Prosperity”: How Artificial Intelligence Usage Impacts Employees’ Innovative Behavior and Proactive Skill Development

**DOI:** 10.3390/bs15040465

**Published:** 2025-04-03

**Authors:** Jin Qian, Jiaxi Chen, Shuming Zhao

**Affiliations:** School of Business, Nanjing University, Nanjing 210093, China; 602022020068@smail.nju.edu.cn (J.Q.); chenjq16@lzu.edu.cn (J.C.)

**Keywords:** artificial intelligence usage, job absorption, AI job replacement anxiety, innovative behavior, proactive skill development, learning goal orientation

## Abstract

As Artificial Intelligence (AI) has become a crucial element in the competitive advantage of enterprises, it is important to understand how to stimulate employees’ creativity and initiative to cope with AI-driven changes. Drawing from the traditional Chinese wisdom of “remaining vigilant while enjoying prosperity” and based on the Conservation of Resources Theory, this study explored the impact of AI usage on employees’ innovative behavior and proactive skill development. The results of a three-stage survey of 350 questionnaires showed that (1) AI usage positively influences employees’ innovative behavior and proactive skill development; (2) job absorption partially mediates the relationship between AI usage and employees’ innovative behavior; (3) AI job replacement anxiety partially mediates the relationship between AI usage and proactive skill development; and (4) employees’ learning goal orientation positively moderates the impact of AI usage on innovative behavior through job absorption and on proactive skill development through AI job replacement anxiety. This study provides insights into how individuals respond to AI-driven changes and offers a novel perspective for developing research on AI usage at the individual level.

## 1. Introduction

As technology advances, the world is undergoing a profound transformation powered by Artificial Intelligence (AI), ushering in a new industrial revolution (Syam & Sharma, 2018). AI, which is capable of autonomous learning and decision-making that simulates human intelligence (Tang et al., 2022), is viewed by leaders across various industries as key to enhancing organizational efficiency and creativity. Stanford University’s AI Research Institute recently reported that the global adoption rate of AI in enterprises has surged from 20% in 2017 to 55% in 2023 (Nestor et al., 2024). However, the widespread adoption of AI, while boosting organizational efficiency, has fueled employee anxiety over job replacement and the demands of adapting to rapidly changing professional environments (Li & Huang, 2020). In traditional Chinese cultures, the concept of “remaining vigilant while enjoying prosperity” emphasizes the importance of preparing for potential crises and uncertainties even during stable times, which is particularly relevant in the context of AI-driven transformation. Although AI provides employees with convenience and innovative resources, it also poses the potential risk of replacement, requiring the acquisition of specialized skills to adapt to AI-integrated work environments (Zhang et al., 2023). Faced with this dual impact, employees are not only required to adopt an “enjoying prosperity” strategy to effectively harness AI-enabled innovative resources and maximize its technical advantages but also to employ a “remaining vigilant” strategy to proactively develop skills to cope with the resource depletion associated with AI usage. Jarrahi (2018) highlighted that innovative behavior and proactive skill development are essential for achieving effective and functional human–AI collaboration, thereby enhancing the effectiveness of AI applications in organizations. Therefore, the primary aim of this study is to explore the impact of AI usage on employees’ innovative behavior and proactive skill development.

According to the Conservation of Resources (COR) theory, the same situation can lead to both resource accumulation and resource depletion (Liu et al., 2020). AI usage refers to the integration of AI technology in the workplace, involving interactions among personnel, technology, organizational structure, and environment (Yu et al., 2023). Ackerman and Kanfer (2020) pointed out that the automation of routine and standardized tasks can significantly alleviate employees’ cognitive load, thereby enabling them to allocate their mental resources to more cognitively demanding and innovative activities, ultimately resulting in enhanced productivity and positive organizational outcomes. However, the pervasive implementation of AI has been shown to significantly constrain employee engagement in decision-making processes and task execution, consequently diminishing their perceived social presence and organizational belongingness (Tu et al., 2023), which in turn triggers AI job replacement anxiety and leads to destructive disclosure effects (Tong et al., 2021). In COR theory, individuals are motivated to acquire, maintain, and protect resources (such as time, energy, and social support). Furthermore, it posits that the acquisition of initial resources may facilitate the acquisition of additional resources, thereby establishing a resource gain spiral (Hobfoll, 1989). By reducing distractions and repetitive tasks, AI could effectively enhance employees’ job absorption and strengthen their innovative resources reservation, thereby motivating their innovative behaviors to further accelerate resource accumulation at both individual and organizational levels (Amabile, 1988; Binnewies et al., 2007). Resource acquisition fosters positive psychological states (Hobfoll, 1989), while potential resource loss may paradoxically yield adaptive outcomes (Hobfoll, 2002). AI job replacement anxiety could trigger individuals to adaptively focus on future resource losses, motivating them to proactively develop new skills to mitigate such risks (Hobfoll, 1989) and cope with associated challenges (Strauss & Parker, 2018).

Learning goal orientation refers to an individual tendency to acquire knowledge and develop abilities through continuous learning, particularly when tackling challenging tasks (Dweck, 2014). Employees with strong learning goal orientation usually exhibit higher intrinsic work motivation and possess greater self-regulatory resources (G. Xu & Wang, 2023), which have a significant impact on how they utilize and allocate limited resources in the context of AI-driven changes. Specifically, employees with a high learning goal orientation tend to perceive technological changes as opportunities and are more willing to adopt emerging technologies for sustained resource acquisition and accumulation (Verma & Singh, 2022). Consequently, they will be able to focus on innovative tasks and goals within AI-integrated work settings, thereby fostering increased innovative performance and creative outcomes (Zhang et al., 2023). Additionally, they also tend to prioritize personal growth and long-term career development and are more sensitive to discrepancies between their current skill set and future workplace requirements (Vandewalle et al., 2001). As a result, they are more likely to experience more job replacement anxiety, which, in turn, drives them to invest more effort into proactively learning new skills (Dweck, 1986). Therefore, this study posits that learning goal orientation may be a crucial boundary condition in understanding how employees adapt to and benefit from AI usage in the workplace.

While existing studies have explored the impact of AI usage on employee attitudes and behaviors (Ackerman & Kanfer, 2020), limited attention has been given to how employees strategically navigate the opportunities and challenges posed by AI proliferation, proactively balancing its benefits and drawbacks to effectively adapt to AI-driven organizational changes. Integrating the wisdom of “remaining vigilant while enjoying prosperity” from traditional Chinese culture with COR theory, this study examines the mechanisms and boundary conditions through which AI usage influences employees’ innovative behavior and proactive skill development. Overall, this study contributes to the AI and the organizational behavior literature by shifting the focus from passive adaptation to proactive strategies, integrating a temporal–cultural lens into the COR theory, and emphasizing the role of individual differences in AI adaptation. By addressing both immediate and long-term adaptation challenges, this research offers a more comprehensive framework for understanding how employees navigate the evolving AI landscape. By examining these dynamics, this research contributes to a more nuanced understanding of AI adaptation processes and provides practical insights for organizations navigating AI-driven transformations. The following sections further elaborate on the study’s theoretical framework, methodology, findings, and implications.

## 2. Theoretical Basis and Hypotheses

### 2.1. The Relationship Between AI Usage and Employees’ Innovative Behavior and Proactive Skill Development

AI refers to the technology that can understand and learn from external data, continuously upgrading itself to achieve specific task objectives (Haenlein & Kaplan, 2019). A high level of AI usage indicates that a substantial portion of an employee’s daily work is completed by AI (Tang et al., 2022). Although AI enhances individuals’ efficiency and innovative potential, it also poses risks and challenges for their future development (Kellogg et al., 2020). To this end, employees should fully recognize the dual change in resources brought by AI (Liang et al., 2022). By adopting the strategies of “enjoying prosperity” and “remaining vigilant”, they can actively embrace the benefits of technological changes while proactively addressing potential losses to manage both current and future resources.

In accordance with the “enjoying prosperity” strategy, the effective utilization of AI can stimulate employees’ innovative behavior. Innovative behavior is defined as the actions of individuals who generate novel and creative ideas or solutions and strive to implement these ideas in their daily work (Scott & Bruce, 1994). According to the COR theory, the adequacy of resources is crucial in fostering innovative behavior (Scott & Bruce, 1994). Firstly, AI can enable employees to obtain additional cognitive, energy, and time resources (Jia et al., 2024). AI is characterized by high reliability and accuracy (Balasubramanian et al., 2022) and excels in highly procedural and standardized tasks (Huang & Rust, 2018; Tang et al., 2022). With its support, employees are able to free themselves from the initial, structured, low-level, and mundane tasks of the daily work (Jia et al., 2024) and can engage in more creative, analytical, and decision-making tasks (Chan et al., 2021). For instance, AI can automatically screen resumes based on talent criteria (Kim & Heo, 2021), assist hotel guests with heavy luggage (H. Wang et al., 2022), and reduce employees’ physical and repetitive labor tasks (Autor et al., 2003). By conserving and accumulating resources, employees can invest more in addressing complex and challenging issues, thereby enhancing their innovative behavior. Secondly, Haenlein and Kaplan (2019) highlight that AI can interpret and learn from external data and utilize these insights to flexibly achieve specific goals. AI can also provide employees with timely and effective information (Lee et al., 2015), efficiently process complex information and data analysis, and offer decision-making suggestions when needed (Brynjolfsson & Mitchell, 2017). Consequently, AI can allow them greater control over their work methods, progress, and choices, thereby improving their intrinsic motivation and innovative self-efficacy (Ren et al., 2015). Finally, the effective use of AI enables employees to better apply their knowledge to key tasks, including autonomous decision-making and information integration within the organization (Brougham & Haar, 2018). By doing so, employees will have a greater range of emotional and psychological resources to cope with complex tasks, thus leading to more innovative behaviors (Gu & Peng, 2010). Accordingly, we propose the following:

**Hypothesis** **1:** *AI usage has a positive impact on employee innovative behavior.*

In accordance with the “remaining vigilant” strategy, proactive skill development is crucial for employees to navigate AI-related challenges. Proactive skill development refers to employees proactively acquiring knowledge, skills, and abilities relevant to future work (Claes & Ruiz-Quintanilla, 1998), which reflects their efforts in accumulating resources and adapting to AI-related changes and risks (Strauss & Parker, 2018). It is suggested that by 2030, approximately 800 million jobs worldwide are expected to be redesigned or gradually disappear due to the integration of AI (Manyika & Lund, 2017). According to the COR theory, work is a vital resource for individuals (Selenko et al., 2013). When employees perceive a potential job replacement or recognize that their current skills may not suffice for future job requirements, they will promptly activate resource investment mechanisms to proactively address the potential loss (Hobfoll, 1989). Firstly, job positions replaced by AI may pose a risk of job resource loss and lead to job insecurity for employees during the AI deployment process (Presbitero & Teng-Calleja, 2022). In response, employees may engage in proactive skill development behaviors to acquire the skills and resources needed for future job markets, thereby addressing future risks and challenges (Strauss & Parker, 2018). Secondly, AI-supported tasks exhibit a high degree of skill diversity (Verma & Singh, 2022), introducing new challenges with cross-disciplinary, advanced, and complex skill requirements for employees (Yang & Qiu, 2020). This increase in job skill demands may lead employees to worry that AI will gradually reduce opportunities for tasks that use their current specialized knowledge and skills, causing them to focus adaptively on possible resource loss (Zhu et al., 2021). To remain competitive in the labor market, employees may engage in proactive skill development to improve their skill resources (Strauss & Parker, 2018). Accordingly, we propose the following:

**Hypothesis** **2:** *AI usage has a positive impact on employee proactive skill development.*

### 2.2. The Mediating Role of Job Absorption

Job absorption is a positive and work-related psychological state characterized by a complete immersion in one’s work (Bakker & Schaufeli, 2008). AI is capable of performing tasks autonomously and systematically without the intervention of humans (Glikson & Woolley, 2020). According to the COR theory, the use of AI can help employees preserve cognitive, energy, and time resources (Hobfoll, 1989). With adequate resources, employees can not only experience less stress but also be more motivated to engage in their tasks, which can enhance their job absorption. Additionally, AI can assist employees in handling physical and repetitive tasks (Autor et al., 2003), allowing them to break free from monotonous and tedious work (Zhao et al., 2019). By effectively reducing work-related stress (Ackerman & Kanfer, 2020) and providing a more meaningful work experience (Jia et al., 2024), AI can also foster more favorable task characteristics and work environments conducive to job absorption. Consequently, a higher level of AI usage may increase employee job absorption. Accordingly, we propose the following:

**Hypothesis** **3:** *AI usage has a positive impact on employee job absorption.*

Innovative behavior is defined as a proactive activity that involves generating novel ideas, seeking necessary support, and implementing these ideas (Scott & Bruce, 1994). According to the COR theory, proactive innovation is closely related to the availability of personal resources (Hobfoll, 2002). Firstly, job absorption provides employees with the resources necessary for innovation. When employees are fully absorbed in their work, they will experience a diminished sense of time and emotional concerns, resulting in feelings of ease, enjoyment, and peak performance (Amada, 1991). According to Oldham and Cummings (1996), such a state can facilitate the acquisition of resources required to solve higher-level issues, thereby triggering a resource-gain spiral and increasing the likelihood of engaging in innovative behavior. Secondly, innovative behavior is an investment characterized by high uncertainty and risk (Carmeli & Schaubroeck, 2007). Through job absorption, resources that can be acquired can alleviate the anxiety associated with the risks and uncertainties of innovative behavior. As a result of this reduction in perceived risk, employees may be more inclined to pursue bold innovations. Finally, the use of AI in complex and challenging tasks has been shown to enhance employee motivation and creativity (Chae & Choi, 2018), leading to the generation of more innovative ideas. Therefore, AI usage can help employees acquire additional resources, allowing them to concentrate on higher-level tasks and invest these resources in innovative behaviors. Accordingly, we propose the following:

**Hypothesis** **4:** *AI usage has a positive impact on employee innovative behavior through job absorption.*

### 2.3. The Mediating Role of AI Job Replacement Anxiety

AI job replacement anxiety refers to the apprehension and fear stemming from the potential or observed replacement of human roles by AI (Li & Huang, 2020). Firstly, the unemployment or bleak employment prospects resulting from AI advancements can trigger significant anxiety regarding job replacement among employees. Artificial Intelligence’s advantages in performing specific tasks are steadily growing, and it is increasingly evident that AI will take on more job duties in the future (Huang & Rust, 2018; Liang et al., 2022). As a result of this trend, employees are likely to perceive AI as a potential threat to their jobs, leading to widespread job insecurity (Yam et al., 2023). Secondly, AI has permeated nearly all industries, and its demonstrated accuracy and efficiency have been widely recognized by businesses. It is therefore expected that AI will displace human workers in an increasing number of fields in the future (P. Xu & Xu, 2020). Consequently, it will be more difficult for employees to obtain new employment opportunities in other organizations, thereby exacerbating concerns about their future employment (H. Lin et al., 2024). Accordingly, we propose the following:

**Hypothesis** **5:** *AI usage has a positive impact on employees’ AI job replacement anxiety.*

According to the COR theory, individuals under stress may invest additional effort in acquiring valuable future resources to offset current resource losses (Hobfoll, 1989). AI job replacement anxiety exposes employees to the pressure of potential future resource losses (Li & Huang, 2020). This type of anxiety is more in line with facilitative anxiety, which can drive employees to exert greater effort and persistence in acquiring new professional skills (Y. Y. Wang & Wang, 2022). As a result, employees with a higher level of AI job replacement anxiety are more likely to invest extra effort, seek new knowledge, and develop new skills to secure resources beneficial for their future career development. In essence, greater AI job replacement anxiety correlates with increased effort invested in proactively learning new knowledge and skills. Accordingly, we propose the following:

**Hypothesis** **6:** *AI usage has a positive impact on employee proactive skill development through AI job replacement anxiety.*

### 2.4. The Moderating Role of Learning Goal Orientation

Learning goal orientation refers to an individual’s tendency to develop their abilities through acquiring new skills, mastering new situations, and learning from practical experiences (Dweck, 1986). It reflects a strong desire for the ability to adapt to new environments and for the enhancement of self-efficacy, both of which affect behavior in stressful situations (Vandewalle, 1997). According to the Conservation of Resources Theory, the initiation of proactive behaviors in individuals is closely tied to the resources they possess. Abundant resources facilitate individuals’ adaptation to stress and organizational change (Hobfoll, 1989), enabling them to acquire new resources through strategic resource investments, thereby fostering subsequent resource growth (Hobfoll, 2002). This study posits that learning goal orientation can amplify the positive effects of AI usage on job absorption. Firstly, learning goal orientation is a stable personal trait and an essential internal resource (Ten Brummelhuis & Bakker, 2012). Employees with a high learning goal orientation tend to be focused on learning and progress, willing to undertake challenging tasks, and more likely to invest effort in acquiring skills and knowledge in an AI-enhanced work environment. With the assistance and augmentation provided by AI, such employees can enhance the availability of work resources necessary for tackling complex, higher-level tasks, thereby reducing the need to engage in fundamental tasks themselves. As a result of the improved protection and optimization of these key resources, employees are more likely to concentrate on their tasks, thus enhancing job absorption. Moreover, individuals with a high learning goal orientation are more likely to perceive AI-related challenges as opportunities for acquiring knowledge and personal growth. When faced with difficulties, they are inclined to invest additional effort and adopt a proactive approach to solving the problem (Dweck, 2014). This proactive engagement can further enhance their focus on specific tasks and objectives, resulting in a higher level of job absorption. Conversely, employees with a low learning goal orientation are more likely to perceive AI usage as resource depletion. This perception can impede their ability to master and utilize AI effectively, leading to internal resource allocation conflicts and, subsequently, lower job absorption. Accordingly, we propose the following:

**Hypothesis** **7:** *Learning goal orientation positively moderates the relationship between AI usage and job absorption.*

Integrating Hypothesis 4 and Hypothesis 7, we further propose the following:

**Hypothesis** **8:** *Learning goal orientation moderates the indirect effect of AI usage on employee innovative behavior through job absorption.*

Learning goal orientation is generally regarded as a specific cognitive framework that individuals adopt when facing pressure. Individuals with a strong learning goal orientation are highly motivated to learn and progress, to focus on skill development, and to respond positively to challenging situations to improve their abilities (Dweck, 1986). This study posits that learning goal orientation will strengthen the impact of AI usage on job replacement anxiety. Individuals with a high learning goal orientation embrace challenging tasks and have long-term career plans and goals (Vandewalle et al., 2001). Therefore, they are more sensitive to the reduction in employment opportunities requiring human professional skills as a result of the widespread adoption of Artificial Intelligence. They are also more likely to perceive the gap between their current skills and the future demands of the workplace, leading to job replacement anxiety through self-assessment (Bandura, 1986). In contrast, employees with a low learning goal orientation lack long-term career planning and struggle to accurately anticipate potential future risks and challenges. They are more likely to be content with the status quo and fail to perceive potential career threats, resulting in lower job replacement anxiety. Accordingly, we propose the following:

**Hypothesis** **9:** *Learning goal orientation positively moderates the relationship between AI usage and AI job replacement anxiety.*

Integrating Hypothesis 5 and Hypothesis 9, we further propose the following:

**Hypothesis** **10:** *Learning goal orientation moderates the indirect effect of AI usage on proactive skill development through AI job replacement anxiety.*

**Figure 1:**
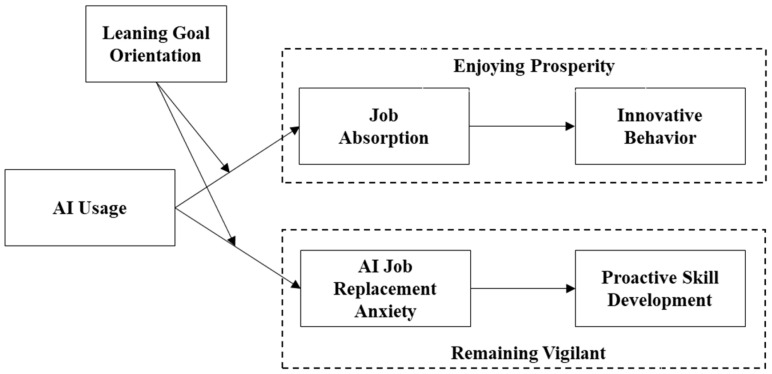
Theorical model.

The theorical model is depicted in Figure 1.

## 3. Methods

### 3.1. Sample Collection

From December 2023 to March 2024, we collected data in three stages across nine high-tech companies using AI in five Chinese cities: Nanjing, Hangzhou, Suzhou, Changzhou, and Wuxi. To ensure methodological validity, we adopted a purposive sampling approach, selecting organizations accredited as National High-Tech Enterprises. During the preparatory phase, with the support of alumni enterprises, we contacted the leaders of potential target companies to explain the study’s purpose and procedures in detail. Ultimately, nine companies that met the criteria agreed to participate in the survey. After obtaining approval from company leaders, we requested that each company assign an HR manager to facilitate the survey process. The HR managers were responsible for distributing the informed consent forms and questionnaire to employees through a random sampling approach. Employees were provided the opportunity to complete the survey during work hours, with assurances that their responses would remain strictly confidential and anonymous. To mitigate common method bias, data collection was conducted in three waves, with each wave spaced seven days apart. Participants’ responses were matched using the last five digits of their phone numbers.

To ensure questionnaire validity, we defined AI usage at the beginning of the first questionnaire as “the extent to which employees use AI technologies or tools in their work, such as intelligent robots, big data, voice recognition, large language models like ChatGPT-4, and machine learning”. After reading this definition, respondents were asked a screening question: “Have you ever used AI-related technologies in your work?” Those who answered “yes” proceeded to the main questionnaire, while those who answered “no” were deemed ineligible and excluded. To ensure the credibility, we embedded an attention check question: “Please select ‘uncertain’ for this question”. Participants that did not follow this instruction were considered invalid. In the first stage, we assessed AI usage, learning goals, and basic information about employees, with 523 questionnaires returned, 451 of which were valid (an 86% response rate). Participants who passed the screening question and provided valid answers in the first stage completed the second stage questionnaire seven days later, which measured job absorption and AI job replacement anxiety. This stage yielded 437 questionnaires, of which 402 were valid (a 92% response rate). Following both stages, respondents who provided valid answers completed the third-stage questionnaire seven days later, which measured innovative behavior and proactive skill development. A total of 395 questionnaires were collected, of which 350 were ultimately valid (an 89% response rate).

Among the respondents, 53.4% were male and 46.6% were female. The majority were aged between 30 and 39, accounting for 42.6% of the sample. In terms of the education level, 45.7% had an associate degree or lower, 39.7% had a bachelor’s degree, 12% had a master’s degree, and 2.6% had a doctoral degree. All respondents had some experience using AI, with the majority (40.3%) having two to three years of experience. Regarding job positions, 44% were staff-level employees, 41.1% were junior managers, 14% were middle managers, and 0.9% were senior managers. Additionally, 61.4% had been in their current positions for less than five years.

### 3.2. Measurement

To ensure reliability and validity, we universally adopted established scales. To accurately translate English scales into Chinese, we strictly followed a translation-back-translation procedure. Except for control variables, all constructs were measured using a 5-point Likert scale (1 = “completely disagree”; 5 = “completely agree”).

#### 3.2.1. Artificial Intelligence Usage

Artificial Intelligence usage was measured with the three-item scale adopted by Tang et al. (2022). A sample item was “I used artificial intelligence to perform most of my job functions”. Cronbach’s α was 0.739.

#### 3.2.2. Job Absorption

Job absorption was measured with the six-item scale adopted by Schaufeli et al. (2002). A sample item was “When I am working, I forget everything else around me”. Cronbach’s α was 0.905.

#### 3.2.3. AI Job Replacement Anxiety

AI job replacement anxiety was measured with the three-item scale adopted by Y. Y. Wang and Wang (2022). A sample item was “I am concerned that the widespread use of humanoid robots will result in job losses”. Cronbach’s α was 0.799.

#### 3.2.4. Innovative Behavior

Innovative behavior was measured with the nine-item scale adopted by Ng and Lucianetti (2016). A sample item was “I created new ideas to improve my work”. Cronbach’s α was 0.847.

#### 3.2.5. Proactive Skill Development

Proactive skill development was measured with the three-item scale adopted by Claes and Ruiz-Quintanilla (1998). A sample item was “Over the past few weeks, to what extent have you developed skills that may be needed in the future?” Cronbach’s α was 0.772.

#### 3.2.6. Learning Goal Orientation

Learning goal orientation was measured with the six-item scale adopted by Vandewalle (1997). A sample item was “I frequently read materials related to my work to improve my ability”. Cronbach’s α was 0.860.

#### 3.2.7. Control Variables

According to Tang et al. (2022), the duration of AI usage may affect employees’ perceptions and attitudes toward AI. Age, tenure, and gender were identified as potential factors affecting proactive behavior by Strauss and Parker (2018), who observed that older and longer-tenured employees may exhibit a lower level of proactive behavior. Previous research also revealed gender differences in future orientation. Additionally, Ng and Lucianetti (2016) highlighted that individuals with greater job responsibilities and higher education levels tend to have more opportunities to innovate and disseminate ideas at work. Therefore, for this study, we selected age, gender, education level, job position, tenure, and duration of AI usage as control variables.

## 4. Results

### 4.1. Confirmatory Factor Analysis

Firstly, we conducted a Confirmatory Factor Analysis (CFA) using Mplus 7.4 to test the discriminant validity of the variables. The results in Table 1 indicated that the six-factor model fit the data better than alternative models (χ^2^/df = 1.99, RMSEA = 0.053, CFI = 0.916, TLI = 0.907, SRMR = 0.046), which suggests that the variables had strong discriminant validity and could be used for further data analysis.

### 4.2. Common Method Bias Test

To reduce the impact of Common Method Bias (CMB), we employed procedural controls, including anonymous surveys, clear instructions in the questionnaire, reverse-coded items, and a three-wave measurement process. Statistically, we used the Harman’s single-factor test with SPSS 26.0 to check for CMB. The analysis revealed that the first factor accounted for 31.673% of the total variance, well below the 50% threshold, indicating that CMB was not a significant concern in this study (Fuller et al., 2016).

### 4.3. Correlation Analysis

In the present study, we conducted basic correlation analysis using SPSS 26.0. The results in Table 2 showed that AI usage is significantly positively correlated with job absorption, AI job replacement anxiety, innovative behavior, and proactive skill development (r = 0.433, r = 0.478, r = 0.469, r = 0.447, *p* < 0.01). Job absorption is significantly positively correlated with innovative behavior (r = 0.457, *p* < 0.01), and AI job replacement anxiety is significantly positively correlated with proactive skill development (r = 0.533, *p* < 0.01). Learning goal orientation is significantly positively correlated with AI usage, job absorption, and AI job replacement anxiety (r = 0.407, r = 0.397, r = 0.404, *p* < 0.01). These results provide preliminary support for the hypotheses.

### 4.4. Hypothesis Testing

As recommended by L. Wang and Preacher (2015), we used path analysis and the bootstrap resampling method for hypothesis testing. The analysis results are presented in Table 3. In Table 3, path a1 denotes the impact of AI usage on job absorption, while path a2 indicates the effect of AI usage on AI job replacement anxiety. Path b1 shows the influence of job absorption on employee innovative behavior, and path b2 illustrates the impact of AI job replacement anxiety on proactive skill development. Path c1 signifies the direct effect of AI usage on employee innovative behavior, and path c2 represents the direct effect of AI usage on proactive skill development. Path d1 demonstrates the moderating effect of learning goal orientation on path a1, and path d2 shows the moderating effect of learning goal orientation on path a2. Paths e1 to e6 and f1 to f6 denote the effects of the control variables.

From Table 3, it is evident that AI usage significantly enhances employee innovative behavior (β = 0.296, *p* < 0.001) and proactive skill development (β = 0.264, *p* < 0.001). Therefore, Hypotheses 1 and 2 are supported. Furthermore, job absorption has a significant positive effect on employee innovative behavior (β = 0.148, *p* < 0.01), and the indirect effect of AI usage on employee innovative behavior through job absorption is 0.058 (*p* < 0.05), with a 95% bootstrap confidence interval of [0.015, 0.114], which does not include zero. Therefore, Hypotheses 3 and 4 are supported. Additionally, AI job replacement anxiety significantly boosts proactive skill development (β = 0.292, *p* < 0.001), and the indirect effect of AI usage on proactive skill development through AI job replacement anxiety is 0.130 (*p* < 0.01), with a 95% bootstrap confidence interval of [0.071, 0.202], not including zero. Hence, Hypotheses 5 and 6 are supported.

**Figure 2:**
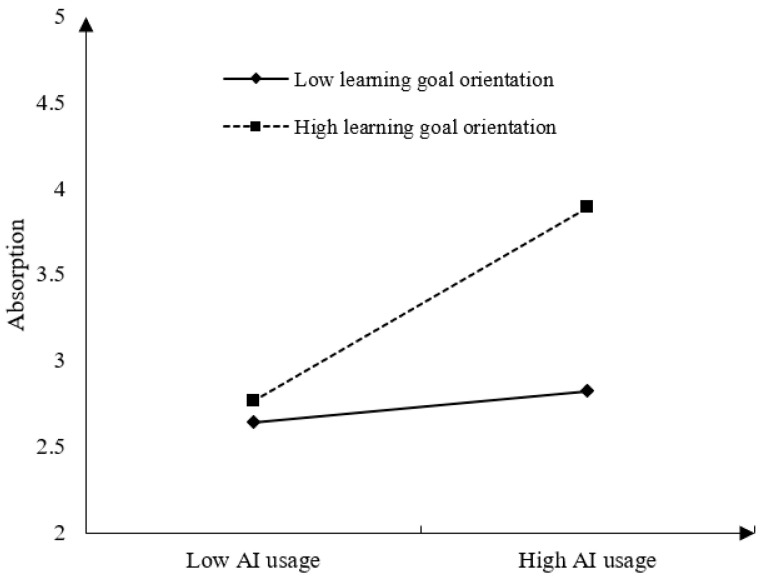
The moderation effect of learning goal orientation on the relationship between AI usage and job absorption.

In the moderation analysis, as depicted in Table 3 and Figure 2, employees’ learning goal orientation significantly and positively moderates the relationship between AI usage and job absorption (β = 0.248, *p* < 0.001). Simple slope tests indicate that when employees have a high learning goal orientation (+1SD), AI usage has a stronger positive impact on job absorption (simple slope = 0.584, *p* < 0.001). Conversely, when employees have a low learning goal orientation (−1SD), AI usage has a weaker positive impact on job absorption (simple slope = 0.382, *p* < 0.001), with a significant difference between the two conditions (diff = 0.257, *p* < 0.05). Thus, Hypothesis 7 is supported, and the moderation effect is illustrated in Figure 2.

**Figure 3:**
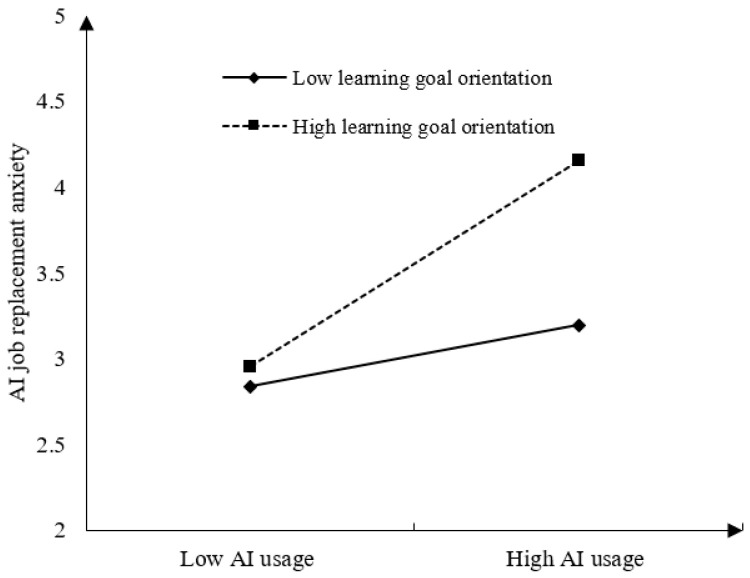
The moderation effect of learning goal orientation on the relationship between AI usage and AI job replacement anxiety.

Furthermore, as depicted in Table 3 and Figure 3, employee learning goal orientation also significantly and positively moderates the relationship between AI usage and AI job replacement anxiety (β = 0.219, *p* < 0.001). Simple slope tests demonstrate that when employees have a high learning goal orientation (+1SD), AI usage has a stronger positive impact on AI job replacement anxiety (simple slope = 0.612, *p* < 0.001). On the other hand, when employees have a low learning goal orientation (−1SD), AI usage has a weaker positive impact on AI job replacement anxiety (simple slope = 0.275, *p* < 0.01). The difference is also significant (diff = 0.336, *p* < 0.001). Therefore, Hypothesis 9 is supported, and the moderation effect is depicted in Figure 3.

Table 4 presents the results of the moderated mediation analysis and bootstrap testing for the indirect effects. From Table 4, it is evident that when employees have high learning goal orientation (+1SD), AI usage has a relatively stronger indirect effect on employee innovative behavior through job absorption (indirect = 0.087, *p* < 0.05, 95% CI = [0.023, 0.163]). Conversely, when employees have low learning goal orientation (−1SD), the indirect effect of AI usage on employee innovative behavior through job absorption is weaker (indirect = 0.030, *p* < 0.05, 95% CI = [0.007, 0.064]), with a significant difference between the two (diff = 0.057, *p* < 0.05, 95% CI = [0.017, 0.106]). Therefore, Hypothesis 8 is supported. Meanwhile, when employees have high learning goal orientation (+1SD), AI usage has a relatively stronger indirect effect on proactive skill development through AI job replacement anxiety (indirect = 0.179, *p* < 0.001, 95% CI = [0.105, 0.264]). Conversely, when employees have low learning goal orientation (−1SD), the indirect effect of AI usage on proactive skill development through AI job replacement anxiety is weaker (indirect = 0.080, *p* < 0.01, 95% CI = [0.032, 0.149]), with a significant difference between the two (diff = 0.098, *p* < 0.001, 95% CI = [0.063, 0.150]). Therefore, Hypothesis 10 is supported.

**Figure 4:**
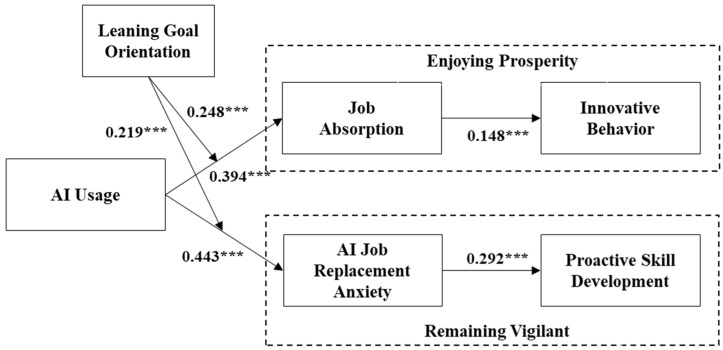
The results of path analysis. Notes: *** *p* < 0.001.

To explain the conceptual model and delineate the hypothesized relationships between constructs, we further demonstrated the results of path analysis in Figure 4.

## 5. Discussion

Based on the COR theory, this study conducted a three-stage survey among employees in AI-deployed enterprises to explore the impact of AI usage on employee innovative behavior and proactive skill development through job absorption and AI job replacement anxiety, as well as the moderating role of learning goal orientation in these relationships. The empirical analysis results indicate that (1) AI usage positively influences employee innovative behavior and proactive skill development; (2) job absorption partially mediates the relationship between AI usage and employee innovative behavior; (3) AI job replacement anxiety partially mediates the relationship between AI usage and proactive skill development; and (4) employees’ learning goal orientation positively moderates the impact of AI usage on innovative behavior through job absorption and on proactive skill development through AI job replacement anxiety.

### 5.1. Theoretical Contributions

Firstly, this study provides a more proactive perspective on employee adaptation to AI, challenging the predominantly passive narratives in existing research. Prior studies have largely examined AI’s impact on employees in a deterministic manner, emphasizing how AI influences work characteristics (Verma & Singh, 2022), enhances productivity through data processing (Tong et al., 2021), or induces aversion due to its “black box” nature (Mahmud et al., 2022). However, limited attention has been given to employees’ agency in shaping their adaptation strategies. By identifying job absorption and AI job replacement anxiety as key cognitive mechanisms, this study empirically demonstrates that employees do not merely react to AI passively but actively engage in adaptation processes. The findings reveal that employees who experience higher job absorption are more likely to integrate AI into their work, while those who experience greater AI job replacement anxiety may proactively seek skill development to maintain their employability. This dual-path mechanism extends the literature on employee–AI interactions beyond the traditional acceptance-based paradigm and highlights the importance of proactive adaptation strategies.

Secondly, this study advances the COR theory by integrating a temporal and cultural perspective to explain how AI simultaneously generates resource gains and depletion. Existing research primarily examines the impact of AI usage from a present-focused perspective, which limits its ability to account for employees’ strategies in managing future uncertainties and resource fluctuations (Yin et al., 2024). The COR theory posits that individuals strive to acquire and protect resources to avoid future resource loss (Hobfoll, 1989). This study builds on this principle by incorporating the cultural wisdom of “remaining vigilant while enjoying prosperity” from traditional Chinese thought, emphasizing that employees must not only leverage AI-enabled work and innovation resources in the present but also anticipate and mitigate potential long-term resource depletion, such as job instability and skill obsolescence. The findings demonstrate that employees who effectively manage this dual-resource dynamic—capitalizing on AI’s benefits while preemptively addressing its risks—exhibit better adaptation outcomes. This contribution refines the COR theory by demonstrating how proactive resource management strategies can shape employees’ long-term adaptation to AI, bridging short-term benefits with long-term sustainability. Furthermore, it underscores the role of cultural mindsets in influencing employees’ responses to technological change, offering a novel perspective in cross-cultural organizational research.

Thirdly, this study identifies learning goal orientation as a critical boundary condition in AI adaptation, enriching the understanding of individual differences in AI usage. While previous studies have focused on organizational factors such as AI readiness (Yin et al., 2024), organizational support (Chatterjee et al., 2021), and leadership styles (Wijayati et al., 2022), relatively little attention has been paid to individual characteristics that shape AI adaptation. Given that AI is not inherently “intelligent” but requires effective human collaboration to maximize its potential (Wilson & Daugherty, 2018), understanding the role of individual traits is crucial. This study empirically validates that employees with a higher learning goal orientation are more likely to engage in job absorption and proactively develop new skills, thereby strengthening their ability to adapt to AI-driven changes. Conversely, employees with lower learning goal orientation exhibit weaker adaptation responses. These findings extend the boundary conditions of AI adaptation research and highlight the necessity of considering individual differences in shaping AI’s effectiveness within organizations.

### 5.2. Practical Implications

Firstly, employees should maintain a positive attitude and adapt to AI-driven changes. As AI rapidly advances, companies across various industries are accelerating AI deployment. In the future, human employees will increasingly delegate mechanical and cognitive tasks to AI, enabling them to focus on emotional and innovative tasks (Huang & Rust, 2018). As the “main actors” in this technological transformation, employees should be more proactive rather than confrontational to the risks and challenges brought about by AI. Specifically, they can adopt the strategies of “enjoying prosperity” and “remaining vigilant” identified in this study. On the one hand, they should exploit the potential of AI to improve their creativity as well as their creative self-efficacy. On the other hand, they should address the potential threats posed by AI and transform stress into motivation by proactively building AI-related skills rather than passively avoiding them.

Secondly, managers should accelerate the implementation of human resource management (HRM) practices that align with the organizational AI transformation strategy. Specifically, it is particularly important for managers to consider the existing employees’ AI-related skills and value orientations when preparing for AI deployment. They should then develop personalized training programs to enhance employees’ AI skills and stimulate their innovative capabilities. Additionally, managers should pay special attention to employees with lower learning goal orientation, developing psychological counseling and career planning programs to help them establish the correct value orientation. The optimization of organizational HRM practices can ensure that everyone’s efforts align with the strategic direction of AI-driven organizational change, thus making HRM a true strategic partner of the organization (Zhao et al., 2021).

Finally, organizations should fully recognize that the wave of digital transformation is unstoppable and that the advantages of AI can only be realized with human collaboration (Jarrahi, 2018; Wilson & Daugherty, 2018). Therefore, organizations should promptly develop a corporate culture that aligns with AI-driven changes. For example, organizations can implement a fault-tolerant mechanism to mitigate the effects of human–AI collaboration failures on employee acceptance and effective use of AI (Webber et al., 2019). As opposed to merely mandating AI usage, organizations should engage employees throughout the entire AI deployment process, helping them understand how the new technology works and facilitating its integration (Han et al., 2023). In addition, organizations should focus on job redesign in order to clarify AI-related responsibilities (He et al., 2023), assist employees in utilizing new technology for higher-level tasks, and guide them in effectively adjusting to new technologies. By fostering a human-centered and harmonious coexistence between humans and machines, organizations can fully unleash the potential value of AI.

### 5.3. Limitations and Directions for Future Research

This study has several limitations. Firstly, the research sample consists primarily of high-tech enterprises, which may limit the generalizability of the AI application scenarios due to industry-specific characteristics. To further validate the study’s findings, future research should consider using samples from a broader range of industries, such as service, transportation, and healthcare. Secondly, although we collected data in three stages, causal relationships between the constructs may not have been fully established. Future research could design scenario experiments or longitudinal studies to further explore the mechanisms behind AI usage. Thirdly, we only considered one of the three dimensions of job engagement—the role of job absorption—which may limit the understanding of how employees’ overall engagement influences AI adaptation. Given that vigor and dedication capture different aspects of employees’ physical and psychological investment in work, future research could explore their roles individually or examine job engagement as a whole to provide a more comprehensive perspective in similar contexts. Fourthly, as AI advances, the nature of human–AI collaboration may change. Future studies could consider factors such as individual intrinsic learning motivation (Y. M. Wang et al., 2022), career orientation (T. C. Lin et al., 2013), and leadership-related factors (Chowdhury et al., 2022) to develop a more robust theoretical model. Finally, while self-reported measures provide critical insights into employees’ subjective experiences of innovative behavior, future research could adopt methods such as supervisor evaluations or peer assessments to provide more objective evaluations of employees’ innovative behaviors to reduce potential self-report bias.

## 6. Conclusions

The efficiency brought by AI cannot be fully realized without employees’ proactive and creative behavior. Therefore, in facing the opportunities and challenges posed by AI, employees have to respond to AI-driven changes and manage the dialectical relationship between resource acquisition and depletion more effectively. While extensive research has explored AI’s impact on employee attitudes and behaviors, limited attention has been given to how employees strategically adapt to AI-driven changes, proactively leveraging its benefits while mitigating its risks. Drawing on the COR theory and traditional Chinese cultural wisdom, this study highlights a dual adaptation mechanism. On one hand, employees can adopt an “enjoying prosperity” strategy to embrace AI’s advantages and harness AI-enabled innovative resources. On the other hand, employees can adopt a “remaining vigilant” strategy to address job replacement anxiety and channel this pressure into proactive skill development for long-term career growth. These findings could offer valuable insights for scholars, practitioners, and enterprise managers to understand how employees effectively adapt to AI-driven organizational change more deeply.

## Figures and Tables

**Table 1:** The results of Confirmatory Factor Analysis.

Model	χ^2^	df	χ^2^/df	RMSEA	CFI	NFI	SRMR
AIUSE; ABS; JRA; IB; PSD; LGO	776.169	390	1.99	0.053	0.916	0.907	0.046
AIUSE; ABS; JRA; IB + PSD; LGO	876.392	395	2.22	0.059	0.896	0.885	0.049
AIUSE; ABS + JRA; IB + PSD; LGO	1410.301	399	3.53	0.085	0.781	0.761	0.078
AIUSE + ABS + JRA; IB + PSD; LGO	1557.972	402	3.88	0.091	0.750	0.729	0.080
AIUSE + ABS + JRA + IB + PSD; LGO	1771.093	404	4.38	0.098	0.704	0.682	0.082
AIUSE + ABS + JRA + IB + PSD + LGO	2275.201	405	5.62	0.115	0.595	0.565	0.098

Note: AIUSE = Artificial Intelligence usage; ABS = job absorption; JRA = AI job replacement anxiety; IB = innovative behavior; PSD = proactive skill development; LGO = learning goal orientation.

**Table 2:** Matrix of mean, standard deviation, and correlation coefficient of variables.

Variables	1	2	3	4	5	6	7	8	9	10	11	12
1. Gender	-											
2. Age	0.003	-										
3. Education Level	−0.042	−0.494 **	-									
4. Duration of AI Usage	−0.028	−0.001	−0.067	-								
5. Job Position	−0.038	0.743 **	−0.300 **	−0.012	-							
6. Tenure	−0.001	0.850 **	−0.354 **	0.047	0.723 **	-						
7. AI Usage	0.114 *	−0.011	−0.068	−0.008	0.015	−0.040	(0.739)					
8. Job Absorption	0.135 *	0.009	−0.047	−0.025	−0.006	−0.033	0.433 **	(0.905)				
9. AI Job Replacement Anxiety	0.089	−0.009	−0.028	0.012	−0.011	−0.002	0.478 **	0.465 **	(0.799)			
10 Innovative Behavior	0.161 **	−0.071	0.003	−0.035	−0.051	−0.094	0.469 **	0.457 **	0.522 **	(0.847)		
11. Proactive Skill Development	0.102	−0.053	−0.024	−0.078	−0.025	−0.009	0.447 **	0.386 **	0.533 **	0.432 **	(0.772)	
12. Learning Goal Orientation	0.040	−0.055	0.022	0.001	0.017	−0.026	0.407 **	0.397 **	0.404 **	0.368 **	0.369 **	(0.860)
M	0.466	2.217	3.583	3.240	1.717	3.180	3.437	3.341	3.536	3.509	3.562	3.465
SD	0.500	1.099	0.968	1.057	0.732	1.379	0.877	0.965	0.962	0.766	0.900	0.770

Note: ** *p* < 0.01, * *p* < 0.05.

**Table 3:** The results of path analysis.

Path	Estimate	S.E.
The path of “enjoying prosperity”		
a1	0.394 ***	0.057
b1	0.148 **	0.050
c1	0.296 ***	0.047
Mediation effect a1 × b1	0.058 *	0.024
Total effect a1 × b1 + c1	0.354 ***	0.040
d1	0.248 ***	0.043
e1	0.101	0.077
e2	−0.010	0.057
e3	0.002	0.038
e4	−0.020	0.028
e5	−0.015	0.052
e6	−0.030	0.053
The path of “remaining vigilant”		
a2	0.443 ***	0.060
b2	0.292 ***	0.058
c2	0.264 ***	0.052
Mediation effect a2 × b2	0.130 ***	0.033
Total effect a2 × b2 + c2	0.394 ***	0.050
d2	0.219 ***	0.043
f1	0.035	0.072
f2	−0.169 *	0.083
f3	−0.048	0.042
f4	−0.080 *	0.038
f5	−0.032	0.079
f6	0.117 *	0.057

Note: *** *p* < 0.001, ** *p* < 0.01, * *p* < 0.05.

**Table 4:** The results of moderated mediation analysis.

Path	Learning Goal Orientation (−1SD/+1SD)	Effect	S.E.	95% Confidence Interval
AI Usage → Job Absorption → Innovative Behavior	Low	0.030 *	0.015	[0.007, 0.064]
	High	0.087 *	0.035	[0.023, 0.163]
	Difference	0.057 *	0.023	[0.017, 0.106]
AI Usage → AI Job Replacement Anxiety → Proactive Skill Development	Low	0.080 **	0.028	[0.032, 0.149]
	High	0.179 ***	0.041	[0.105, 0.264]
	Difference	0.098 ***	0.026	[0.063, 0.150]

Notes: *** *p* < 0.001, ** *p* < 0.01, * *p* < 0.05.

## Data Availability

The raw data supporting the conclusions of this article will be made available by the authors, without undue reservation.
